# Effects of Self-Compassion and Mindfulness Interventions on Mental Health and Work-Related Outcomes Among Japanese Workers: Randomized Controlled Trial

**DOI:** 10.2196/79991

**Published:** 2026-03-17

**Authors:** Takumu Kurosawa, Koichiro Adachi, Ryu Takizawa

**Affiliations:** 1 Department of Clinical Psychology, Graduate School of Education, The University of Tokyo, 7-3-1, Hongo, Bunkyo-ku, Tokyo, 113-0033, Japan, 81 03-3812-2111

**Keywords:** self-compassion, mindfulness, mobile apps, digital health, occupational health, mental health, randomized controlled trial, RCT, work performance

## Abstract

**Background:**

Mental health problems among workers are a significant global concern, leading to substantial economic losses, particularly due to presenteeism. While mindfulness and self-compassion practices have shown promise in improving psychological well-being and occupational outcomes, traditional interventions often require a high time commitment. Low-intensity digital interventions remain underexplored, especially in the context of occupational health.

**Objective:**

This study aimed to develop and evaluate the effectiveness of a smartphone-based self-care app that enables independent practice of mindfulness and self-compassion without facilitator support. Specifically, it assessed the effects of self-compassion meditation (SCM) and mindfulness meditation (MM) on mental health and work-related outcomes among nonclinical Japanese workers.

**Methods:**

This open-label, 3-arm randomized controlled trial recruited 300 working adults in Japan, who were randomly assigned to SCM (n=101), MM (n=100), or a waitlist control group (n=99). Participants in the SCM and MM groups engaged in daily guided meditation via a custom-built smartphone app over 4 weeks. Primary outcomes included psychological distress (Kessler Psychological Distress Scale-6) and work performance (World Health Organization Health and Work Performance Questionnaire and the Stanford Presenteeism Scale), while secondary outcomes encompassed cognitive flexibility, self-compassion, perceived stress, work engagement, psychological safety, and self-perceived creativity. Assessments were conducted at baseline, postintervention (4 wk), and 1-month follow-up (8 wk). Recruitment occurred in 2 waves (November 2022-December 2022 and June 2023), with follow-up assessments completed by September 2023.

**Results:**

A total of 300 participants (mean age of 35.44, SD 9.14 y; n=180, 60% female) were randomized. Adherence was high in both intervention groups (mean completed days: SCM 23.30, SD 5.58; MM 22.95, SD 6.25), with an overall dropout rate of 21.4%. No significant group × time interactions were detected for most outcomes, although significant main effects of time were observed for several measures, including work performance and self-compassion, with small effect sizes. Within-group analyses suggested broader improvements in the SCM group, whereas changes in the MM group were more limited. In sensitivity analyses using linear mixed-effects models, a significant group × time interaction was detected for work performance, with a larger pre-post improvement in SCM.

**Conclusions:**

This low-intensity, fully self-guided, smartphone-based meditation program demonstrated good feasibility, as reflected by high adherence and relatively low attrition. Between-group effects were limited; therefore, the findings should be interpreted as preliminary and do not support strong causal claims of intervention superiority. Clinical significance remains unclear in this nonclinical sample and should be evaluated in future studies.

## Introduction

Mental health problems among workers pose a critical challenge with far-reaching implications for the global economy. According to the World Health Organization (WHO), depression and anxiety disorders account for the loss of approximately 12 billion workdays annually, resulting in an annual economic burden of approximately US $1 trillion [1]. A survey of 10,000 workers in Japan identified mental illnesses, stiff shoulders, and lower back pain as health problems affecting job performance. Among these, presenteeism—employees being present at work but unable to perform at full capacity due to mental illness—causes the most significant economic losses [2]. These findings underscore the need for initiatives that improve workers’ mental health and productivity.

Mindfulness and self-compassion are effective approaches for supporting workers’ mental health. These approaches are often categorized within the “third wave” of cognitive-behavioral therapies, which emphasize context- and acceptance-based strategies aimed at altering one’s relationship with internal experiences rather than primarily modifying cognitive content [3]. Mindfulness is the practice of maintaining nonjudgmental awareness of one’s thoughts, emotions, bodily sensations, and surrounding environment in the present moment [4], while self-compassion entails being warm and understanding toward ourselves when we suffer, fail, or feel inadequate, rather than ignoring our pain or flagellating ourselves with self-criticism [5]. Mindfulness practice has been shown to enhance focus at work, self-fulfillment, and sleep quality, while reducing emotional exhaustion, occupational stress, and anxiety [6-8]. It has also been reported to improve job performance, workplace relationships, and creativity [9,10]. Similarly, self-compassion has been found to alleviate stress and burnout and to improve well-being and resilience. However, further research is needed to clarify its specific effects on work-related outcomes [11].

Mindfulness and self-compassion both use meditation as a core intervention strategy and share the goal of fostering nonjudgmental acceptance of one’s experiences. However, self-compassion places additional emphasis on responding to difficulty with self-kindness and a sense of common humanity [12]. Furthermore, mindfulness-based interventions may cultivate self-compassion more implicitly than self-compassion–focused programs, which teach these elements explicitly [13]. Elevated levels of self-compassion have been correlated with more adaptive emotion regulation and enhanced well-being [14], and interventions aimed at increasing self-compassion have proven effective in mitigating self-criticism [15]. Within the employee samples, self-compassion has been linked to job-related outcomes, including performance indicators [16], and its related capacities may contribute to the development of psychologically safer environments and promote learning-oriented behaviors within teams [17]. When implementing short-term, low-intensity interventions for busy workers, it is crucial to select the most effective components. Accordingly, directly comparing mindfulness meditation (MM) and self-compassion meditation (SCM) may help clarify which elements are particularly relevant for improving work-related outcomes.

Traditional mindfulness and self-compassion programs typically consist of 8 weeks of in-person sessions with daily practice, which has been identified as a barrier to participation for busy workers [4,5,18]. In contrast, digital interventions using websites or smartphone apps have been shown to be effective in reducing stress and improving well-being, while requiring less time and cost [19,20]. These low-intensity interventions provide a more accessible alternative for users. Digital interventions exhibit varying levels of human support, ranging from fully self-administered programs to guided formats that incorporate contact with a coach or therapist [21]. Although unguided interventions offer high scalability and low cost, they tend to show lower adherence and reduced efficacy compared to guided approaches [21]. In the context of occupational digital mental health interventions, additional support and increased intensity may improve engagement and outcomes, albeit at the expense of scalability and increased implementation demands [22]. Consequently, engagement with mindfulness-based mobile apps varies widely, underscoring the importance of evaluating feasibility and reporting engagement transparently, especially in fully self-guided delivery [23]. However, despite their growing popularity, these low-intensity digital interventions remain underrepresented in randomized controlled trials (RCTs), underscoring the need for further empirical research. Additionally, the COVID-19 pandemic has accelerated the adoption of online psychological support, emphasizing the importance of developing accessible digital tools to assist workers, especially as remote work becomes increasingly prevalent [24]. In response to this need, this study aims to develop a self-care app that facilitates independent practice of mindfulness and self-compassion and to evaluate its effectiveness using mental health and work-related outcomes through an RCT design.

## Methods

### Study Design

This open-label, 3-arm RCT compared the effects of 2 intervention groups—the SCM and MM groups—with a waitlist control group (WCG). Both intervention groups engaged in guided meditation sessions delivered through a mobile app developed specifically for the study. Further details are provided in the study protocol [25].

### Participants

The inclusion criteria were employees who were (1) working more than 20 hours per week, (2) aged between 18 and 54 years, (3) not on a leave of absence, (4) not business owners or students, and (5) not currently diagnosed with a mental disorder and scoring below 13 on the Kessler Psychological Distress Scale-6 [26]. The smartphone app used in this study was only available on iOS, restricting participation to iPhone users. The study involved 300 workers in Japan via open calls from websites. Participants were required to have basic digital literacy to install and use the smartphone app and to complete web-based questionnaires. The participants were not informed of their group allocation.

### Ethical Considerations

This study was approved by the ethics committee of the University of Tokyo (22-326). The study was registered with the University Hospital Medical Information Network Clinical Trials Registry (UMIN000049466), and the study protocol was published previously [25]. Electronic informed consent was obtained via a web-based form prior to eligibility screening and randomization, and participants could withdraw at any time without penalty. Survey responses and app log data were managed and analyzed using study-generated participant IDs. Identifying information was stored separately from study data; access to the data was restricted to the research team, and the linkage file was stored offline in a secure location. Participants received a gift card worth up to JP ¥10,000 (approximately US $63; JP ¥158=US $1 as of January 11, 2026), depending on completion of study procedures; compensation was not contingent on adherence to the intervention (eg, the number of meditation days completed).

### Procedure

Participants were recruited via open calls during 2 recruitment waves (November 2022-December 2022 and June 2023) through a psychology experiment recruitment website and our laboratory’s website, due to insufficient enrollment in the first wave. Study details and contact information were available on website posters. Interested individuals completed a web form with a screening survey (Kessler Psychological Distress Scale-6) and were screened based on the inclusion criteria. The baseline assessment (T0), the posttreatment assessment at 4 weeks (T1), and the follow-up assessment at 8 weeks (T2) were assessed using self-report questionnaires. Questionnaires at each time point were administered independently of the intervention through an external web-based form, rather than within the app, with survey links distributed to participants via email. For the SCM and MM groups, T2 (8 wk) represents the 1-month follow-up after the 4-week intervention period. As assessments were scheduled relative to each participant’s intervention timeline, the exact calendar dates varied by participant; however, all follow-up (T2) assessments were completed by September 2023. No significant methodological changes were implemented after trial commencement; any deviations from the protocol regarding outcome measures are described in the “Methods” section.

### Randomization and Blinding

After obtaining informed consent and screening participants, those who met the eligibility criteria were randomly assigned to 1 of the 3 groups (1:1:1) via a computerized blocked randomization scheme (block size=15). This randomization was conducted independently by research staff, and participants were immediately assigned to either the intervention (SCM or MM) or WCG. The intervention commenced approximately 1 week after allocation, while the waitlist group received no specific instructions and waited for 4 weeks. Participants received group-specific instructions via automated email. As the participants followed a meditation guide, blinding was not possible, thus making this an open-label RCT. The instructions were sent via automated emails, and although the participants could infer their group, they were not explicitly informed of their assignment.

### Interventions

The SCM and MM courses were delivered through a smartphone app developed by the research team. Participants assigned to the SCM and MM groups used the app to engage in guided meditation once daily for 4 weeks. Each course comprised 3 modules that integrated guided self-help meditation and psychoeducation. The guided meditations in weeks 1 and 2 were identical across both courses: a 7-minute breathing meditation in week 1 and a 6-minute short body scan in week 2. In weeks 3 and 4, SCM participants practiced a 12-minute loving-kindness meditation focused on self-kindness and common humanity, while MM participants engaged in a 12-minute breath, sound, and body meditation. The guided meditations were adapted from scripts developed by the University of California, Los Angeles, Mindful Awareness Research Center and modified for a Japanese audience. Psychoeducation covered topics such as stress responses, mindfulness, and self-compassion and was provided as the studies were accessible via the app at any time. Weekly email reminders encouraged participation, though no minimum time commitment was required. Meditation practice was systematically recorded by the app through data logging, and engagement metrics were derived from these logs. The intervention was fully automated and self-directed, with no support from a human instructor or therapist. Participants could view their completed practice days and total practice time within the app.

We did not instruct participants to use the app in a specific location and did not collect data on the settings where sessions were completed (eg, home vs workplace); participants were advised to practice in a quiet, private environment without interruptions. Participants in the WCG received no intervention during the 4-week period; afterward, they were allocated to either the SCM or MM course for an additional 4 weeks of guided meditation. Because WCG participants were offered access to an intervention after the 4-week waiting period for ethical reasons, they did not remain in a controlled condition during weeks 4 to 8 and were therefore not included in the 8-week (T2) follow-up analyses.

### Outcome Measures

This study aimed to investigate the efficacy of a brief, self-administered smartphone-based meditation program, focusing on self-compassion and mindfulness, in enhancing workers’ mental health and work-related functioning. Therefore, the primary outcomes were defined as (1) psychological distress, which is a direct target of the intervention; and (2) work performance (presenteeism), a pragmatic occupational outcome. As secondary outcomes, we assessed potentially modifiable psychological processes and related indicators (eg, cognitive flexibility, self-compassion, perceived stress, and work engagement) to complement the evaluation of changes in mental health and work-related outcomes. Although daily physiological measures (including heart rate and heart rate variability) were planned in the protocol, they are not reported in this paper because this report focuses on psychological and work-related outcomes; these physiological outcomes will be reported separately.

### Primary Outcomes

The primary outcomes were psychological distress and work performance, assessed at 3 time points: baseline (T0), posttreatment at 4 weeks (T1), and follow-up at 8 weeks (T2). Psychological distress was measured using the Japanese version of the Kessler Psychological Distress Scale-6 [26,27], a 6-item instrument designed to measure nonspecific psychological distress (depressive mood and anxiety) over the preceding 30 days (total score range: 0‐24; higher scores indicate greater distress), with good internal consistency in this study (Cronbach α at T0=0.85). A cutoff score of 12/13 on the Kessler Psychological Distress Scale-6 has been proposed for screening severe depressive symptoms [28], and thus, an ethical exclusion criterion of Kessler Psychological Distress Scale-6 ≥13 was established. Work performance was evaluated using one presenteeism item from the Japanese version of the World Health Organization Health and Work Performance Questionnaire (WHO-HPQ) [29,30], which assesses overall work performance over the past 28 days (absolute presenteeism score range: 0‐100; higher scores indicate better performance). Moreover, work inefficiency was assessed using the work impairment score from the Stanford Presenteeism Scale (SPS) [31,32], which captures the extent to which health-related cognitive, emotional, and behavioral difficulties impede task completion and focus (10 items; 1=“always” to 5=“never”; scored such that higher scores indicate greater impairment). This scale exhibited acceptable internal consistency (Cronbach *α*=0.72) in this study. While the protocol specified presenteeism assessed using the WHO-HPQ, this report additionally includes the SPS work impairment score to capture health-related work impairment.

### Secondary Outcomes

The following self-reported questionnaires were administered as secondary outcome measures at each time point: the Cognitive Flexibility Scale (Cronbach *α*=0.86) [33,34], which assesses the ability to generate alternative responses and adapt behavior to changing circumstances (12 items; total score range: 12‐72; higher scores indicate greater cognitive flexibility); the Self-Compassion Scale (SCS; Cronbach *α*=0.94) [35,36], which assesses compassionate self-responding toward oneself across 6 facets (26 items; total score range: 26‐130; higher scores indicate greater self-compassion), including its subscales: self-kindness (Cronbach *α*=0.92), common humanity (Cronbach *α*=0.80), mindfulness (Cronbach *α*=0.79), self-judgment (Cronbach *α*=0.89), isolation (Cronbach *α*=0.84), and overidentification (Cronbach *α*=0.81); the Perceived Stress Scale-14 (Cronbach *α*=0.89) [37-39], which assesses the extent to which situations in the past month are perceived as unpredictable, uncontrollable, and overloaded (total score range: 0‐56; higher scores indicate greater perceived stress); and the Utrecht Work Engagement Scale-9 (Cronbach *α*=0.95) [40-42], which assesses vigor, dedication, and absorption (total score range: 0‐54; higher scores indicate higher engagement), including its subscales: vigor (Cronbach *α*=0.89), dedication (Cronbach *α*=0.84), and absorption (Cronbach *α*=0.88). Additionally, psychological safety was assessed using the Psychological Safety Scale (7 items; Cronbach *α*=0.80) [43,44], which reflects the extent to which individuals can express themselves at work without fear of negative consequences; items are rated from 1 (“not at all”) to 7 (“strongly agree”), yielding total scores of 7 to 49. Creativity (idea generation at work) was assessed using the Self-Perceived Creativity Scale (3 items; Cronbach *α*=0.91) [45], rated from 1 (“do not agree”) to 7 (“agree”), yielding total scores of 3 to 21. For further details on these outcome measures, refer to the study protocol [25].

### Other Measures

Demographic variables were assessed using a questionnaire at baseline (T0). The collected variables included age, gender, employment status, marital status, educational background, and prior meditation experience, including mindfulness practices.

### Sample Size

In the protocol, the sample size was planned based on detecting a small group-by-time interaction effect (Cohen *f*=0.15) for the primary pre-post comparison across the 3 arms, assuming a correlation of 0.50 between repeated measures, with 80% power at a 2-sided *α*=.05 (G*Power; Heinrich Heine University Düsseldorf) [25]. This yielded a required sample size of 37 participants per arm. The protocol, therefore, initially planned to randomize 200 participants to account for expected attrition. During the trial conduct, we observed that a proportion of participants did not complete the baseline assessment following randomization; therefore, recruitment was extended and a total of 300 participants were randomized to ensure an adequate evaluable sample size at key assessment time points and to improve the precision of effect estimates. This target sample size was reflected in the trial registration (UMIN000049466). The increase in sample size was an operational decision made without reference to intervention effects, and no interim efficacy analyses were conducted.

### Statistical Analyses

#### Missing Data and Sensitivity Analysis

Analyses were conducted using available cases at each assessment point. Participants with missing data for a specific analysis were excluded from that analysis, resulting in varying sample sizes across time points. As a sensitivity analysis, linear mixed-effects models (LMMs) were fitted using all available data to assess the robustness of the findings in relation to attrition.

#### Baseline Analyses

Demographic variables and baseline psychological indices were summarized descriptively (mean and SD for continuous variables; n and % for categorical variables) for each group. In accordance with CONSORT-EHEALTH (Consolidated Standards of Reporting Trials of Electronic and Mobile Health Applications and Online Telehealth) guidance (Checklist 1), we did not perform significance testing for baseline group comparisons.

#### Analysis of Preintervention and Postintervention Effects

Changes before and after the intervention or waitlist periods were assessed using 2-tailed paired *t* tests within each intervention group. Effect sizes were calculated as Cohen *d* and interpreted using cutoff values of 0.20, 0.50, and 0.80 for small, medium, and large effects, respectively [38].

#### Analysis of Effect Size in Group Comparisons

To examine preintervention and postintervention changes and pre-post differences between groups, assessment questionnaire results were analyzed using a 2 (time: pre and post) × 3 (group) repeated-measures ANOVA. Effect sizes for ANOVA were reported as generalized η², with cutoff values of 0.01, 0.06, and 0.14 for small, medium, and large effects, respectively [38,46].

#### Analysis of 1-Month Follow-Up

As the waitlist group transitioned to receive an intervention after T1, all T2 follow-up analyses were restricted to participants initially allocated to SCM or MM. The 1-month follow-up scores were analyzed using a series of one-way within-subjects ANOVAs to compare scores within each intervention group across three time points: (1) preintervention, (2) postintervention, and (3) 1-month follow-up. For measures that showed significant time effects, Bonferroni-adjusted pairwise comparisons were conducted to examine changes within each intervention group.

To assess the maintenance of treatment effects and differences between the intervention groups, a 3 (time: pre, post, and follow-up) × 2 (group: SCM and MM) repeated-measures ANOVA was performed. For measures that showed significant time effects, Bonferroni-adjusted pairwise comparisons were conducted to investigate changes across the intervention groups.

#### Sensitivity Analyses Using LMMs

Primary inference in this trial was derived from between-group comparisons (group × time). Within-group tests in the main analyses were conducted as exploratory analyses to describe within-group patterns and should not be interpreted as evidence of causal superiority. As a sensitivity analysis, we reanalyzed the primary and secondary outcomes using LMMs to assess the robustness of the findings. These models included fixed effects for group, time, and their interaction, with a random intercept for participants to account for within-subject correlations. Additionally, variables showing notable baseline imbalances were included as covariates in sensitivity analyses to assess the robustness of the findings. The models were fitted using all available data under a missing-at-random assumption. For the pre-post comparison (T0-T1), analyses included all randomized groups (SCM, MM, and WCG), whereas follow-up analyses (T0-T1-T2) were restricted to SCM and MM because the WCG crossed over after T1.

## Results

### Participant Flow and Sample Characteristics

**Figure 1.**
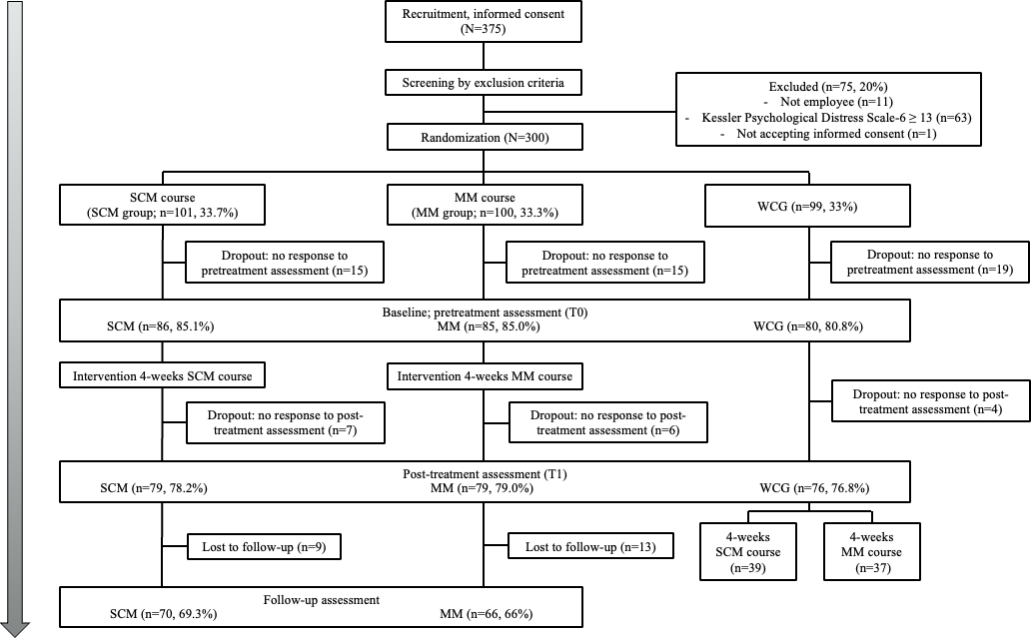
CONSORT (Consolidated Standards of Reporting Trials) flow diagram of participant enrollment, allocation, and follow-up assessments. MM: mindfulness meditation; SCM: self-compassion meditation; WCG: waitlist control group.

In total, 300 participants were randomly allocated to SCM (n=101), MM (n=100), and WCG (n=99; Figure 1). Of these 300 randomized participants, 251 completed the baseline assessment (T0; 83.7%), including 86 (85.1%) in SCM, 85 (85%) in MM, and 80 (80.8%) in WCG, and were included in Table 1. At posttreatment (T1), outcome data were available for 234 participants (78%; SCM: n=79, 78.2%; MM: n=79, 79%; WCG: n=76, 76.8%). At follow-up (T2), outcome data were available for 136 (67.7% of the SCM and MM) participants in the intervention arms (SCM: n=70, 69.7%; MM: n=66, 66%). No important harms or unintended effects (including major technical problems or privacy incidents) were reported to the study team during the trial. Among those who completed the baseline assessment (Table 1), the sample consisted of 60% (n=180) female participants, with a high proportion of regular employees (n=57, 67.1%). The mean age was 35.44 (SD 9.14) years. The groups were generally similar at baseline; however, age and prior meditation experience showed some imbalance across groups. Therefore, age (grand-mean centered) and prior meditation experience were included as covariates in the sensitivity analyses using LMMs (Figure 1).

**Table 1.** Baseline demographic characteristics of participants by group.

Variable	SCM^a^ group (n=86)	MM^b^ group (n=85)	Waitlist group (n=80)
Age (y), mean (SD)	33.96 (8.84)	35.02 (9.65)	37.45 (8.65)
Sex, n (%)			
Female	51 (59.3)	47 (55.3)	52 (65)
Male	34 (39.5)	38 (44.7)	28 (35)
Employment status, n (%)			
Regular	59 (68.6)	57 (67.1)	52 (65)
Nonregular	27 (31.4)	27 (31.8)	28 (35)
Marital status, n (%)			
Single	50 (58.1)	52 (61.2)	35 (43.8)
Married	35 (40.7)	32 (37.6)	45 (56.2)
Educational attainment, mean (SD)	15.70 (1.67)	15.88 (1.72)	15.79 (1.63)
Previous meditation experience, n (%)			
Yes	30 (34.9)	18 (21.2)	15 (18.8)
No	56 (65.1)	66 (77.6)	64 (80)
Psychological distress, mean (SD)	7.05 (4.51)	6.23 (4.96)	6.62 (4.92)
Work performance, mean (SD)	62.79 (18.32)	59.88 (18.16)	60.25 (20.68)
Work inefficiency, mean (SD)	26.46 (5.21)	26.89 (6.02)	26.13 (4.92)
Cognitive flexibility, mean (SD)	47.28 (8.43)	46.01 (8.46)	47.00 (9.11)
Self-compassion, mean (SD)	76.39 (17.43)	74.14 (19.46)	79.85 (18.73)
Self-kindness, mean (SD)	15.74 (4.55)	15.02 (4.89)	16.16 (4.81)
Self-judgment, mean (SD)	15.80 (4.82)	15.79 (4.76)	14.83 (5.26)
Common humanity, mean (SD)	10.89 (3.72)	10.85 (3.76)	11.49 (3.80)
Isolation, mean (SD)	10.81 (4.05)	11.74 (4.04)	10.74 (4.44)
Mindfulness, mean (SD)	12.47 (3.07)	11.88 (3.39)	12.81 (3.18)
Overidentification, mean (SD)	13.52 (3.55)	13.75 (3.86)	13.46 (3.72)
Perceived stress, mean (SD)	28.50 (9.58)	28.75 (10.81)	28.50 (9.47)
Work engagement, mean (SD)	28.41 (13.51)	26.14 (12.30)	27.69 (12.20)
Vigor, mean (SD)	8.64 (4.72)	7.79 (4.51)	8.66 (4.52)
Dedication, mean (SD)	10.62 (4.53)	9.98 (4.10)	10.28 (4.14)
Absorption, mean (SD)	9.29 (4.95)	8.38 (4.41)	8.57 (4.47)
Psychological safety, mean (SD)	35.30 (6.65)	34.29 (7.44)	33.60 (7.93)
Creativity, mean (SD)	12.19 (4.37)	11.67 (4.35)	11.36 (4.09)

a SCM: self-compassion meditation.

b MM: mindfulness meditation.

The rate of participants who dropped out before posttreatment assessment did not differ among groups (*χ^2^*_2_=0.66; *P*=.72). On average, the participants practiced for 23.30 (SD 5.58) days in the SCM group and 22.95 (SD 6.25) days in the MM group during the intervention period. The 2-tailed *t* tests showed no significant differences in the number of practice days between the 2 groups (*t*_154_=0.38; *P*=.71).

### Pre-Post Differences Between the Groups

Table 2 summarizes the mean and SD for each group, along with preintervention and postintervention comparisons using 2-tailed paired *t* tests within each intervention group. In the SCM group, paired *t* tests revealed significant improvements in work performance, cognitive flexibility, self-compassion, common humanity (as measured by the SCS), and psychological safety. Significant reductions were observed in work inefficiency, perceived stress, self-judgment, and overidentification (as measured by the SCS). In the MM group, 2-tailed paired *t* tests showed a significant improvement in common humanity (as measured by the SCS). Table 3 presents the results of between-group comparisons using ANOVA. No significant interaction effect between group and time was found.

**Table 2.** Mean (SD) scores for intervention and control groups at preintervention and postintervention and changes in scores in each intervention group.

		Preintervention, mean (SD)	Postintervention, mean (SD)	*t* test (*df*)	*P* value	Cohen *d*
Psychological distress						
SCM^a^ group		6.68 (4.27)	6.90 (4.40)	0.49 (77)	.62	0.06
MM^b^ group		5.94 (4.92)	6.38 (5.08)	1.05 (77)	.30	0.09
Waitlist group		6.75 (4.76)	7.12 (5.44)	1.14 (73)	.26	0.09
Work performance						
SCM group		63.29 (16.93)	70.00 (15.85)	4.28 (78)	<.001	0.41
MM group		59.37 (17.27)	60.25 (18.81)	0.49 (78)	.62	0.05
Waitlist group		59.87 (21.07)	62.93 (20.58)	1.56 (74)	.12	0.15
Work inefficiency						
SCM group		26.22 (5.04)	24.85 (5.52)	–2.21 (75)	.03	–0.27
MM group		26.78 (5.99)	25.83 (5.81)	–1.34 (72)	.18	–0.14
Waitlist group		26.15 (5.02)	25.81 (6.00)	–1.11 (71)	.27	–0.09
Cognitive flexibility						
SCM group		47.69 (8.18)	49.04 (8.73)	2.27 (71)	.03	0.18
MM group		45.81 (8.49)	45.86 (9.09)	0.11 (76)	.91	0.01
Waitlist group		46.84 (9.31)	46.85 (9.32)	0.26 (73)	.80	0.01
Self-compassion						
SCM group		76.30 (17.94)	81.17 (18.06)	2.86 (65)	.006	0.24
MM group		74.55 (19.27)	77.32 (20.11)	1.91 (71)	.06	0.10
Waitlist group		79.42 (19.11)	79.70 (19.71)	1.33 (70)	.19	0.07
Self-kindness						
SCM group		15.79 (4.66)	16.56 (4.77)	1.68 (73)	.10	0.15
MM group		15.12 (4.91)	15.48 (4.99)	0.80 (77)	.42	0.05
Waitlist group		16.28 (4.90)	16.33 (5.01)	0.15 (75)	.88	0.01
Self-judgment						
SCM group		15.70 (4.96)	14.77 (4.38)	–2.36 (74)	.02	–0.21
MM group		15.68 (4.74)	15.42 (4.92)	–0.83 (77)	.41	–0.05
Waitlist group		15.13 (5.22)	14.69 (4.92)	–1.29 (74)	.20	–0.09
Common humanity						
SCM group		10.67 (3.78)	11.47 (3.98)	2.39 (75)	.02	0.19
MM group		10.97 (3.73)	11.48 (3.75)	2.19 (75)	.03	0.14
Waitlist group		11.56 (3.85)	11.63 (3.81)	0.21 (74)	.83	0.02
Isolation						
SCM group		10.64 (4.10)	10.14 (3.84)	–1.65 (74)	.10	–0.14
MM group		11.51 (3.93)	11.23 (3.81)	–0.85 (78)	.40	–0.07
Waitlist group		10.92 (4.47)	10.91 (4.27)	–0.04 (75)	.97	0.00
Mindfulness						
SCM group		12.38 (3.13)	12.78 (3.53)	1.19 (76)	.24	0.11
MM group		11.94 (3.34)	12.14 (3.55)	0.58 (77)	.56	0.05
Waitlist group		12.80 (3.24)	13.01 (3.16)	0.90 (74)	.37	0.07
Overidentification						
SCM group		13.42 (3.57)	12.74 (3.73)	–2.27 (76)	.03	–0.20
MM group		13.68 (3.89)	13.37 (3.86)	–1.19 (76)	.24	–0.08
Waitlist group		13.57 (3.73)	13.49 (4.12)	–0.31 (73)	.76	–0.02
Perceived stress						
SCM group		28.31 (9.86)	25.19 (8.52)	–2.65 (75)	.01	–0.31
MM group		28.46 (10.69)	27.05 (9.65)	–1.27 (77)	.21	–0.13
Waitlist group		28.99 (9.35)	27.61 (9.54)	–1.39 (67)	.17	–0.11
Work engagement						
SCM group		28.21 (13.55)	29.51 (13.29)	1.32 (73)	.19	0.07
MM group		25.58 (12.04)	26.03 (12.72)	0.52 (78)	.60	0.04
Waitlist group		27.61 (12.07)	28.08 (13.36)	0.79 (73)	.43	0.05
Vigor						
SCM group		8.54 (4.73)	8.96 (4.60)	1.17 (77)	.25	0.08
MM group		7.54 (4.41)	8.04 (4.54)	1.44 (78)	.15	0.11
Waitlist group		8.71 (4.46)	8.84 (4.97)	0.67 (74)	.51	0.05
Dedication						
SCM group		10.59 (4.58)	10.87 (4.55)	1.31 (77)	.19	0.07
MM group		9.84 (4.03)	9.87 (4.30)	0.13 (78)	.90	0.01
Waitlist group		10.28 (4.10)	10.50 (4.43)	0.82 (75)	.42	0.05
Absorption						
SCM group		9.22 (4.95)	9.72 (4.77)	1.27 (75)	.21	0.09
MM group		8.20 (4.35)	8.11 (4.62)	–0.25 (78)	.80	–0.02
Waitlist group		8.60 (4.43)	8.74 (4.78)	0.31 (74)	.76	0.02
Psychological safety						
SCM group		35.40 (6.83)	36.71 (6.73)	2.12 (75)	.04	0.17
MM group		34.34 (7.38)	34.23 (7.79)	–0.18 (78)	.86	–0.01
Waitlist group		33.57 (8.01)	33.80 (9.10)	0.48 (74)	.64	0.04
Creativity						
SCM group		12.17 (4.33)	12.45 (4.46)	1.09 (73)	.28	0.09
MM group		11.55 (4.38)	11.08 (4.26)	–1.18 (77)	.24	–0.10
Waitlist group		11.41 (4.18)	11.65 (4.73)	0.76 (74)	.45	0.06

a SCM: self-compassion meditation.

b MM: mindfulness meditation.

**Table 3.** Group × time interaction effect using ANOVA.

Variable	*F* test (*df*)	*P* value	General η^*2*^
Psychological distress	0.09 (2, 227)	.91	0.000
Work performance	2.67 (2, 230)	.07	0.004
Work inefficiency	0.61 (2, 218)	.54	0.001
Cognitive flexibility	1.70 (2, 220)	.19	0.001
Self-compassion	1.73 (2, 206)	.18	0.001
Self-kindness	0.85 (2, 225)	.43	0.001
Self-judgment	1.19 (2, 225)	.31	0.001
Common humanity	1.36 (2, 224)	.26	0.001
Isolation	0.68 (2, 227)	.51	0.001
Mindfulness	0.15 (2, 227)	.86	0.000
OverIdentification	1.14 (2, 225)	.32	0.001
Perceived stress	1.07 (2, 219)	.34	0.002
Work engagement	0.11 (2, 224)	.90	0.000
Vigor	0.16 (2, 229)	.85	0.000
Dedication	0.28 (2, 230)	.76	0.000
Absorption	0.54 (2, 227)	.58	0.000
Psychological safety	0.99 (2, 227)	.37	0.001
Creativity	1.61 (2, 224)	.20	0.002

As a supplementary analysis, the results of the LMM for repeated measures are shown in Table S1 in Multimedia Appendix 1. A significant group × time interaction was detected for work performance (*F*_2,233_=3.32; *P*=.04; partial η^*2*^=0.028). A simple slope analysis revealed that participants in the SCM group showed a significant change (*t*_237_=3.99; *P*<.001), whereas no significant difference was observed in the MM group (*t*_237_=0.39; *P*=.70) and the WLC group (*t*_237_=1.63; *P*=.11).

After adjusting for age and previous experience with meditation, significant group × time interactions were detected for work performance (*F*_2,230_=3.22; *P*=.04; partial η^*2*^=0.027) and cognitive flexibility (*F*_2,223_=3.13; *P*=.046; partial η^*2*^=0.027). Using simple slope analyses, participants in the SCM group showed a significant change in work performance (*t*_234_ = 3.93; *P*<.001) and cognitive flexibility (*t*_224_ = 2.87; *P*=.004), whereas no significant difference was observed in the MM and WLC groups.

### Follow-Up Analysis

Table 4 summarizes the means and SDs of preintervention, postintervention, and follow-up measures in each intervention group. In the SCM group, significant main effects of time using ANOVA were observed for work performance (*F*_2,138_=6.42; *P* = .002; η*^2^G*=0.029), self-compassion (*F*_2,108_=7.38; *P* <.001; η*^2^G*=0.020), self-judgment (*F*_2,130_=6.19; *P*=.003; η*^2^G*=0.016), common humanity (*F*_2,134_=5.72; *P*=.004; η*^2^G*=0.012), overidentification (*F*_2,130_=3.38; *P*=.04; η*^2^G*=0.008), perceived stress (*F*_2,116_=4.43; *P*=.01; η*^2^G*=0.021), and psychological safety (*F*_2,118_=4.39; *P*=.01; η*^2^G*=0.013). In the SCM group, Bonferroni-adjusted simple comparisons revealed a significant increase in work performance from preintervention to postintervention (*P*<.001) and a significant decrease from postintervention to follow-up (*P*=.03). Significant increases were also observed in self-compassion from preintervention to postintervention (*P*=.03) and from preintervention to follow-up (*P*=.002), common humanity from preintervention to follow-up (*P*=.006), and psychological safety from postintervention to follow-up (*P*=.002). Significant decreases were found in self-judgment from preintervention to follow-up (*P*=.001), and perceived stress from preintervention to follow-up (*P*=.03). In the MM group, significant main effects of time using ANOVA were observed for self-compassion (*F*_2,108_=3.35; *P*=.04; η*^2^G*=0.004) and overidentification (*F*_2,126_=4.28; *P*=.02; η*^2^G*=.007). Bonferroni-adjusted simple comparisons indicated a significant decrease in overidentification from preintervention to follow-up (*P*=.01).

**Table 4.** Mean (SD) at follow-up and repeated-measures analyses across pre, post, and follow-up within the intervention groups.

		Follow-up assessment	Repeated measure ANOVA		
		Mean (SD)	*F* test (*df*)	*P* value	General η*^2^*
Psychological distress					
SCM^a^ group		5.93 (4.03)	1.98 (2, 134)	.14	0.008
MM^b^ group		6.15 (5.21)	0.75 (2, 130)	.47	0.002
Work performance					
SCM group		66.71 (17.00)	6.42 (2, 138)	.002	0.029
MM group		62.42 (18.98)	0.38 (2, 130)	.68	0.002
Work inefficiency					
SCM group		26.21 (4.17)	1.68 (2, 116)	.19	0.013
MM group		25.52 (5.50)	1.02 (2, 114)	.36	0.007
Cognitive flexibility					
SCM group		48.57 (9.70)	2.00 (2, 108)	.14	0.006
MM group		46.86 (8.70)	1.97 (2, 126)	.14	0.004
Self-compassion					
SCM group		81.42 (18.05)	7.38 (2, 108)	<.001	0.020
MM group		77.35 (21.94)	3.35 (2, 108)	.04	0.004
Self-kindness					
SCM group		16.19 (5.12)	1.42 (2, 124)	.24	0.004
MM group		15.69 (5.21)	2.70 (2, 126)	.07	0.005
Self-judgment					
SCM group		14.30 (4.68)	6.19 (2, 130)	.003	0.016
MM group		15.18 (5.06)	2.45 (2, 128)	.09	0.004
Common humanity					
SCM group		11.77 (3.87)	5.72 (2, 134)	.004	0.012
MM group		15.69 (5.21)	2.37 (2, 124)	.10	0.006
Isolation					
SCM group		9.74 (3.69)	2.78 (2, 130)	.07	0.009
MM group		10.83 (3.97)	3.02 (2, 128)	.052	0.007
Mindfulness					
SCM group		13.11 (3.47)	2.70 (2, 134)	.07	0.007
MM group		11.84 (3.53)	0.22 (2, 124)	.80	0.001
Overidentification					
SCM group		12.79 (3.81)	3.38 (2, 130)	.04	0.008
MM group		13.17 (4.14)	4.28 (2, 126)	.02	0.007
Perceived stress					
SCM group		25.03 (9.88)	4.43 (2, 116)	.01	0.021
MM group		26.98 (10.42)	0.56 (2, 126)	.57	0.002
Work engagement					
SCM group		28.91 (13.99)	1.29 (2, 128)	.28	0.002
MM group		25.58 (13.63)	0.45 (2, 128)	.64	0.001
Vigor					
SCM group		8.60 (4.94)	1.44 (2, 136)	.24	0.003
MM group		7.83 (4.65)	0.64 (2, 128)	.53	0.001
Dedication					
SCM group		10.71 (4.63)	1.60 (2, 136)	.21	0.002
MM group		9.73 (4.49)	0.79 (2, 130)	.46	0.002
Absorption					
SCM group		9.60 (5.04)	1.48 (2, 132)	.23	0.002
MM group		8.14 (5.02)	0.26 (2, 130)	.77	0.001
Psychological safety					
SCM group		35.86 (7.37)	4.39 (2, 118)	.01	0.013
MM group		33.79 (7.91)	0.50 (2, 130)	.61	0.001
Creativity					
SCM group		12.19 (4.57)	0.71 (2, 130)	.49	0.001
MM group		11.44 (4.37)	1.23 (2, 128)	.29	0.003

a SCM: self-compassion meditation.

b MM: mindfulness meditation.

In the SCM group, significant main effects of time using LMM were observed for work performance (*F*_2,147_=7.96; *P*<.001; partial η*^2^*=0.098), work inefficiency (*F*_2,141_=3.19; *P*=.04; partial η*^2^*=0.043), cognitive flexibility (*F*_2,135_=3.27; *P*=.04; partial η*^2^*=0.046), self-compassion (*F*_2,134_=8.66; *P*<.001; partial η*^2^*=0.114), self-judgment (*F*_2,148_=6.66; *P*=.002; partial η*^2^*=0.082), common humanity (*F*_2,149_=6.14; *P*=.003; partial η*^2^*=0.076), isolation (*F*_2,148_=3.74; *P*=.03; partial η*^2^*=0.048), overidentification (*F*_2,147_=4.08; *P*=.02; partial η*^2^*=0.053), perceived stress (*F*_2,145_=6.48; *P*=.002; partial η*^2^*=0.082), and psychological safety (*F*_2,143_=3.18; *P*=.04; partial η*^2^*=0.043; Table S2 in Multimedia Appendix 2). In the SCM group, Bonferroni-adjusted simple comparisons revealed a significant increase in work performance from preintervention to postintervention (*P*<.001). Significant increases were also observed in self-compassion from preintervention to postintervention (*P*=.002) and to follow-up (*P*<.001), and in common humanity from preintervention to postintervention (*P*=.04) and to follow-up (*P*=.003). Significant decreases were found in self-judgment from preintervention to postintervention (*P*=.02) and to follow-up (*P*=.002), isolation from preintervention to follow-up (*P*=.02), overidentification from preintervention to follow-up (*P*=.02), and perceived stress from preintervention to postintervention (*P*=.008) and to follow-up (*P*=.008).

In the MM group, significant main effects of time using LMM were observed for self-compassion (*F*_2,133_=4.22; *P*=.02; partial η*^2^*=0.060), self-kindness (*F*_2,144_=3.13; *P*=.047; partial η*^2^*=0.042), common humanity (*F*_2,143_=3.07; *P*=.049; partial η*^2^*=0.041), isolation (*F*_2,145_=3.62; *P*=.03; partial η*^2^*=0.047), and overidentification (*F*_2,144_=3.96; *P*=.02; partial η*^2^*=0.052). Bonferroni-adjusted simple comparisons indicated a significant increase in self-compassion from preintervention to follow-up (*P*=.02) and self-kindness from preintervention to follow-up (*P*=.04). Significant decreases were found in isolation from preintervention to follow-up (*P*=.02) and overidentification from preintervention to follow-up (*P*=.02; Table S2 in Multimedia Appendix 2).

Table 5 summarizes the main effects for time and the interaction effects for group and time. Across all intervention groups, significant main effects for time were found for work performance (*F*_2,268_=3.80; *P*=.02; η*^2^G*=0.009), self-compassion (*F*_2,216_=10.44; *P*<.001; η*^2^G*=0.010), common humanity (*F*_2,258_=7.54; *P*<.001; η*^2^G*=0.008), self-judgment (*F*_2,258_=8.35; *P*<.001; η*^2^G*=0.009), overidentification (in the SCS; *F*_2,256_=6.62; *P*=.001; η*^2^G*=0.007), and perceived stress (*F*_2,242_=4.26; *P*=.02; η*^2^G*=0.008). Bonferroni-adjusted simple comparisons showed that in the intervention group, a significant increase was observed in work performance from preintervention to postintervention (*P*=.01), as well as significant increases in self-compassion from preintervention to postintervention (*P*=.006) and to follow-up (*P*<.001), and in common humanity from preintervention to postintervention (*P*=.02) and to follow-up (*P*=.002). Significant decreases were observed in self-judgment from preintervention to postintervention (*P*=.03) and to follow-up (*P*<.001), in isolation from preintervention to follow-up (*P*=.002), in overidentification from preintervention to postintervention (*P*=.02) and to follow-up (*P*=.002), and in perceived stress from preintervention to follow-up (*P*=.02).

**Table 5.** Main effects for time and interaction effects for group × time using ANOVA.

	Time			Interaction effects		
	*F* test (*df*)	*P* value	General η*^2^*	*F* test (*df*)	*P* value	General η*^2^*
Psychological distress	2.44 (2, 264)	.09	0.004	0.23 (2, 264)	.80	0.000
Work performance	3.80 (2, 268)	.02	0.009	2.32 (2, 268)	.10	0.005
Work inefficiency	2.37 (2, 230)	.10	0.008	0.26 (2, 230)	.77	0.001
Cognitive flexibility	2.48 (2, 234)	.09	0.003	1.53 (2, 234)	.22	0.002
Self-compassion	10.44 (2, 216)	<.001	0.010	0.92 (2, 216)	.40	0.001
Self-kindness	2.22 (2, 250)	.11	0.003	1.68 (2, 250)	.19	0.002
Self-judgment	8.35 (2, 258)	<.001	0.009	1.02 (2, 258)	.36	0.001
Common humanity	7.54 (2, 258)	<.001	0.008	0.32 (2, 258)	.73	0.000
Isolation	5.58 (2, 258)	.004	0.007	0.18 (2, 258)	.84	0.000
Mindfulness	1.57 (2, 258)	.21	0.002	1.12 (2, 258)	.33	0.002
Overidentification	6.62 (2, 256)	.002	0.007	0.95 (2, 256)	.39	0.001
Perceived stress	4.26 (2, 242)	.02	0.008	1.12 (2, 242)	.33	0.002
Work engagement	1.24 (2, 256)	.29	0.001	0.33 (2, 256)	.72	0.000
Vigor	1.94 (2, 264)	.15	0.002	0.10 (2, 264)	.90	0.000
Dedication	1.81 (2, 266)	.16	0.001	0.39 (2, 266)	.67	0.000
Absorption	0.53 (2, 262)	.59	0.001	0.86 (2, 262)	.42	0.001
Psychological safety	2.72 (2, 248)	.07	0.004	1.75 (2, 248)	.18	0.002
Creativity	0.07 (2, 258)	.94	0.000	1.93 (2, 258)	.15	0.002

As a supplementary analysis, the main effects for time and the interaction effects for group × time were examined using LMM (Table S3 in Multimedia Appendix 3). Across all intervention groups, significant main effects for time were found for work performance (*F*_2,292_=5.52; *P*=.004; partial η*^2^*=0.036), self-compassion (*F*_2,267_=3.93; *P*=.02; partial η*^2^*=0.029), self-judgment (*F*_2,293_=3.84; *P*=.02; partial η*^2^*=0.025), and perceived stress (*F*_2,290_=3.35; *P*=.04; partial η*^2^*=0.023). Bonferroni-adjusted simple comparisons showed that in the intervention group, a significant increase was observed in work performance from preintervention to postintervention (*P*=.008), and in self-compassion from preintervention to postintervention (*P*<.001) and follow-up (*P*<.001). Significant decreases were observed in self-judgment from preintervention to postintervention (*P*<.001) and follow-up (*P*=.02), and in perceived stress from preintervention to postintervention (*P*=.006) and follow-up (*P*=.003). A significant group × time interaction was detected for work performance (*F*_2,293_=3.23; *P*=.04; partial η*^2^*=0.022). A simple slope analysis revealed that participants in the SCM group showed a significant increase from preintervention to postintervention (*t*_301_= 3.88; *P*<.001), whereas no other significant differences were observed.

After adjusting for age and previous experience with meditation, all outcomes that were statistically significant in the unadjusted analyses remained significant with respect to the main effect of time. In addition, cognitive flexibility also showed a significant main effect of time (*F*_2,277_=3.09; *P*=.047; partial η*^2^*=0.022). Bonferroni-adjusted simple comparisons showed that in the intervention group, a significant increase was also observed in cognitive flexibility from preintervention to follow-up (*P*=.046). A significant group × time interaction remained for work performance (*F*_2,291_=3.16; *P*=.04; partial η*^2^*=0.021). A simple slope analysis revealed that participants in the SCM group showed a significant increase from preintervention to postintervention (*t*_298_=3.83; *P*<.001), whereas no other significant differences were observed.

## Discussion

### Principal Findings

This study aimed to develop a self-care smartphone app that enables individuals to independently practice mindfulness and self-compassion without the support of a facilitator, and to evaluate its effectiveness using psychological and work-related outcomes. Participants were randomized to SCM, MM, or WCG, and intervention effects were evaluated in an RCT.

SCM participants demonstrated improvements in work performance, cognitive flexibility, self-compassion (especially common humanity), and psychological safety, along with reductions in perceived stress, work inefficiency, self-judgment, and overidentification. In contrast, MM participants only showed improvement in common humanity. No significant group × time interaction was observed in psychological outcomes. However, sensitivity analyses using LMMs revealed a significant group × time interaction for work performance, indicating a relatively greater improvement in the SCM group. Longitudinal analyses revealed that the SCM group maintained several improvements at follow-up, particularly in self-compassion, common humanity, and stress reduction. The MM group showed limited effects, mainly in overidentification. Taken together, within-group analyses suggested improvements in several psychological and work-related outcomes in the SCM group, while changes in the MM group were more limited. Significant reductions were also found in perceived stress, work inefficiency, self-judgment, and overidentification. The next section discusses these findings in detail, including their strengths, limitations, and implications for future research and applied settings.

A comparison of psychological indicators before and after the intervention in the intervention groups revealed that, in the SCM group, work performance, cognitive flexibility, self-compassion, self-esteem, and psychological safety improved compared to preintervention levels, while perceived stress, work inefficiency, self-judgment, and overidentification significantly decreased. In the MM group, a significant increase was observed only in the common humanity subscale of self-compassion. These findings are consistent with previous studies on self-compassion interventions via smartphone apps. For example, a 7-day intervention targeting young people in New Zealand also reported improvements in self-compassion [47]. Similarly, Beshai et al [48] reported that a 4-week guided self-help program (Mind-OP), which combined mindfulness, self-compassion, and goal-setting exercises and included video and audio guidance, resulted in a significant reduction in perceived stress compared to an active control group. This study implemented a 4-week, easy-to-follow guided meditation program without the assistance of a facilitator, targeting Japanese workers, and similarly demonstrated an improvement in self-compassion. Few studies on psychological support for workers have reported intervention effects that extend beyond mental health [11]. This study is one of the few to show that even a short-term self-help intervention among workers can have positive effects on workplace-related outcomes, such as improved work performance, enhanced psychological safety, and reduced productivity loss.

This study used an RCT design to examine the effects of guided meditation practiced individually, using a rigorous approach that compared 2 intervention groups—SCM and MM groups—with a control group. However, when comparing the intervention groups with the control group, no significant interaction effects between intervention and time were observed. One possible reason for the lack of intervention effects relative to the control group is the low intensity of the intervention, which may have resulted in only modest effects. Digital interventions targeting anxiety and depressive symptoms are generally known to yield small to moderate effect sizes [49]. Additionally, the shorter 4-week duration of this fully self-guided intervention, relative to the typical 8-week format of established programs, may account for the limited between-group effects.

Spijkerman et al [50] also pointed out a limitation of psychological interventions in populations with relatively high baseline mental health, similar to participants in this study. Specifically, they noted that individuals without clinical levels of depression or anxiety tend to have lower baseline scores on psychological symptom measures, resulting in limited scope for improvement compared to clinical populations. This may result in smaller effect sizes due to floor effects. In particular, previous studies on online self-compassion interventions have reported small effects on anxiety symptoms and no significant effects on depressive symptoms when compared to active control groups [48].

Moreover, previous research has suggested that therapist-guided interventions are more effective for treating depression than fully self-guided programs [51]. Given that this study used a completely self-directed format without any therapist contact, participants may have found it difficult to independently acquire the necessary skills. This could partly account for the limited improvement, especially in depressive symptoms. In this intervention, loving-kindness meditation was implemented as a form of meditation that incorporates elements of self-compassion. Loving-kindness meditation is also introduced as an optional practice during the day-long retreat in the Mindfulness-Based Stress Reduction program and is recognized as part of the broader mindfulness framework [52]. This type of meditation is designed to cultivate feelings of warmth and benevolence toward oneself and others, including all beings.

Neff and Dahm [53] argued that self-compassion can be more effectively cultivated through explicit rather than implicit methods. The inclusion of loving-kindness meditation, which explicitly integrates components of self-compassion, may have contributed to the effects observed in this intervention over standard mindfulness practices. This may be particularly relevant for workers who are highly self-critical, as the practice of self-compassion could have helped improve their perceived work performance.

Despite the notable findings, this study has several limitations. Specifically, no significant differences were observed in the magnitude of change between the intervention and control groups from preintervention to postintervention. This limitation may be attributed to the relatively low intensity of our intervention program, which may have produced smaller effect sizes insufficient to demonstrate statistically significant group differences.

In terms of feasibility, the study demonstrated better retention and adherence than previous research reporting dropout rates exceeding 50% [54]. Several factors may have contributed to this, including a brief and clearly structured app-based intervention that lasted approximately 6 to 12 minutes per session and allowed participants to complete it at their convenience in terms of time and location. Additionally, weekly email reminders were used. Recruitment via an online crowdsourcing platform—where participants may have approached the intervention as a structured “task”—and the provision of compensation (which was not contingent on the number of meditation days) may have further encouraged engagement. We did not collect or report detailed app usage analytics, such as time-of-day, changes in use intensity over time, or module-level engagement; consequently, total practice time could not be summarized. Given the 1-session-per-day design with a fixed session type and duration (except for interruptions), variability in dose was primarily reflected in the number of completed practice days. Future studies should prospectively capture richer log-based metrics, such as continuity patterns and total practice time, to better characterize feasibility and inform intervention optimization. To enhance intervention effectiveness while maintaining feasibility, future studies could extend the practice period beyond 4 weeks without changing daily practice duration or combine meditation with other intervention techniques (modules). Given the typical trade-off between intervention intensity and accessibility, future research should focus on developing interventions that remain accessible and user-friendly for working adults while improving therapeutic outcomes.

Separately, work performance was assessed using brief self-report measures (a single WHO-HPQ presenteeism item and the SPS work impairment score) to minimize participant burden in this fully self-guided digital trial. Nevertheless, single-item and self-reported measures may be less sensitive to change and may be susceptible to reporting biases, potentially diminishing our ability to detect effects. Future studies should consider incorporating more comprehensive and/or objective indicators of productivity, such as supervisor-rated performance or administrative and behavioral metrics, where feasible.

Furthermore, we could not confirm improvement in depressive symptoms, which was one of our main outcome measures for mental health. It has been noted that unresolved trauma memories may emerge during self-compassion practice, representing a potential risk of such training. For participants with trauma memories practicing at home, it is recommended to limit sessions to about 10 to 15 minutes per day [12]. Therefore, to reduce risks in our fully self-care intervention format, we implemented a low-intensity intervention that could be completed in a short time and targeted only workers with relatively high baseline health status. However, as previous research has indicated, preventive interventions targeting healthy individuals tend to show smaller effects [50]. Future research could incorporate risk reduction strategies, such as regular follow-ups by facilitators based on participants’ circumstances. This approach would potentially allow participation from workers experiencing moderate stress levels, enabling investigations not only into prevention effects for relatively healthy workers but also into supportive effects for workers currently experiencing mental health difficulties.

Finally, the mechanisms by which the intervention improved work performance remain insufficiently understood. Currently, few studies have examined the detailed mechanisms of the relationship between self-compassion practice and work performance. In previous research on mindfulness, a similar psychological intervention, and work performance, mindfulness practice has been shown to enhance attention functions and positively affect major domains of human functioning, including cognition, emotion, behavior, and physiology, thereby improving work performance [10]. Good et al [10] suggest that improved cognitive function and cognitive flexibility may allow sustained attention to work even in environments where attention is depleted or work is performed discontinuously. Additionally, Kirk et al [55], in a study examining emotional regulation in meditation practitioners using economic decision-making games, reported that meditation practitioners activate different brain regions during emotional regulation compared to nonmeditators, suggesting the possibility of rational engagement with others even in emotional situations. Furthermore, higher levels of self-compassion are associated with reduced fear of failure and an increased tendency to try again after failure [56,57]. Based on these findings, future research could enhance understanding of the intervention’s mechanisms by incorporating measures of cognitive function, brain function measurements, and psychological constructs related to failure and goal setting.

### Conclusions

The intervention program was designed to enable nonclinical working adults to practice self-care meditation using a smartphone app without facilitator involvement. The study findings support the feasibility of delivering a brief, low-burden, fully self-guided meditation program incorporating self-compassion elements in occupational settings, with participants able to practice at times and locations of their choosing. However, given the limited between-group effects observed across most outcomes, the improvements noted should be considered preliminary and do not substantiate strong causal claims regarding the superiority of the intervention. Clinical significance remains unclear in this nonclinical sample and should be evaluated in future research. These findings inform the development of scalable, low-burden digital self-care tools for occupational mental health and highlight the need for further studies to evaluate clinical significance.

## Supplementary material

10.2196/79991 Multimedia Appendix 1 Group × time interaction effect using linear mixed-effects models (LMMs).

10.2196/79991 Multimedia Appendix 2 Mean (SD) at follow-up and repeated-measures analyses across pre, post, and follow-up within the intervention groups.

10.2196/79991 Multimedia Appendix 3 Main effects for time and interaction effects for group × time using linear mixed-effects models (LMMs).

10.2196/79991 Checklist 1 CONSORT-EHEALTH checklist [58].

## Abbreviations

CONSORT-EHEALTH: Consolidated Standards of Reporting Trials of Electronic and Mobile Health Applications and Online Telehealth
LMM: linear mixed-effects model
MM: mindfulness meditation
RCT: randomized controlled trial
SCM: self-compassion meditation
SCS: Self-Compassion Scale
SPS: Stanford Presenteeism Scale
WCG: waitlist control group
WHO: World Health Organization
WHO-HPQ: World Health Organization Health and Work Performance Questionnaire
