# Supplementary material for: Effects of Self-Compassion and Mindfulness Interventions on Mental Health and Work-Related Outcomes Among Japanese Workers: Randomized Controlled Trial
Source: J Med Internet Res. 2026 Mar 17;28:e79991. doi: 10.2196/79991 (PMC12994763; doi:10.2196/79991)
Supplement: Multimedia Appendix 1 [file jmir-v28-e79991-s001.docx]

Appendix Table 1. Group × Time interaction effect using LMM

| Variable | ICC | F value | df1 | df2 | *P*-value | partial *η*^2^ |
| --- | --- | --- | --- | --- | --- | --- |
| Psychological Distress | 0.66 | 0.18 | 2 | 231 | .83 | 0.002 |
| Work Performance | 0.62 | 3.32 | 2 | 233 | .04 | 0.028 |
| Work Inefficiency | 0.87 | 0.87 | 2 | 224 | .42 | 0.008 |
| Cognitive Flexibility | 0.81 | 2.30 | 2 | 226 | .10 | 0.020 |
| Self-Compassion | 0.85 | 2.21 | 2 | 213 | .11 | 0.020 |
| Self-Kindness | 0.78 | 0.94 | 2 | 231 | .39 | 0.008 |
| Self-Judgment | 0.78 | 1.46 | 2 | 231 | .23 | 0.012 |
| Common Humanity | 0.77 | 1.37 | 2 | 231 | .26 | 0.012 |
| Isolation | 0.74 | 0.81 | 2 | 232 | .45 | 0.007 |
| Mindfulness | 0.73 | 0.13 | 2 | 234 | .88 | 0.001 |
| Over-Identification | 0.77 | 1.43 | 2 | 231 | .24 | 0.012 |
| Perceived Stress | 0.60 | 1.22 | 2 | 227 | .30 | 0.011 |
| Work Engagement | 0.86 | 0.25 | 2 | 227 | .78 | 0.002 |
| Vigor | 0.80 | 0.13 | 2 | 232 | .88 | 0.001 |
| Dedication | 0.85 | 0.38 | 2 | 233 | .68 | 0.003 |
| Absorption | 0.80 | 0.77 | 2 | 230 | .46 | 0.007 |
| Psychological Safety | 0.72 | 1.15 | 2 | 234 | .32 | 0.010 |
| Creativity | 0.75 | 1.87 | 2 | 232 | .16 | 0.016 |
