# Supplementary material for: Effects of Self-Compassion and Mindfulness Interventions on Mental Health and Work-Related Outcomes Among Japanese Workers: Randomized Controlled Trial
Source: J Med Internet Res. 2026 Mar 17;28:e79991. doi: 10.2196/79991 (PMC12994763; doi:10.2196/79991)
Supplement: Multimedia Appendix 2 [file jmir-v28-e79991-s002.docx]

Appendix Table 2. Mean (SD) at follow-up and repeated-measures analyses across pre, post, and follow-up within the intervention groups

|  | | Follow-up assessment | LMM | | | | | |
| --- | --- | --- | --- | --- | --- | --- | --- | --- |
|  |  | Mean | ICC | F value | df1 | df2 | *P-*value | Partial *η*^2^ |
| Psychological Distress | SCM group | 5.93 (4.03) | 0.57 | 2.00 | 2 | 147 | .14 | 0.026 |
|  | MM group | 6.15 (5.21) | 0.59 | 0.37 | 2 | 146 | .69 | 0.005 |
| Work Performance | SCM group | 66.71 (17.00) | 0.54 | 7.96 | 2 | 147 | <.001 | 0.098 |
|  | MM group | 62.42 (18.98) | 0.69 | 0.48 | 2 | 148 | .62 | 0.006 |
| Work Inefficiency | SCM group | 26.21 (4.17) | 0.37 | 3.19 | 2 | 141 | .04 | 0.043 |
|  | MM group | 25.52 (5.50) | 0.42 | 1.16 | 2 | 146 | .32 | 0.016 |
| Cognitive Flexibility | SCM group | 48.57 (9.70) | 0.75 | 3.27 | 2 | 135 | .04 | 0.046 |
|  | MM group | 46.86 (8.70) | 0.77 | 1.79 | 2 | 145 | .17 | 0.024 |
| Self-Compassion | SCM group | 81.42 (18.05) | 0.78 | 8.66 | 2 | 134 | <.001 | 0.114 |
|  | MM group | 77.35 (21.94) | 0.88 | 4.22 | 2 | 133 | .02 | 0.060 |
| Self-Kindness | SCM group | 16.19 (5.12) | 0.74 | 2.01 | 2 | 146 | .14 | 0.027 |
|  | MM group | 15.69 (5.21) | 0.82 | 3.13 | 2 | 144 | .047 | 0.042 |
| Self-Judgment | SCM group | 14.30 (4.68) | 0.71 | 6.66 | 2 | 148 | .002 | 0.082 |
|  | MM group | 15.18 (5.06) | 0.83 | 2.38 | 2 | 145 | .10 | 0.032 |
| Common Humanity | SCM group | 11.77 (3.87) | 0.78 | 6.14 | 2 | 149 | .003 | 0.076 |
|  | MM group | 15.69 (5.21) | 0.77 | 3.07 | 2 | 143 | .049 | 0.041 |
| Isolation | SCM group | 9.74 (3.69) | 0.70 | 3.74 | 2 | 148 | .03 | 0.048 |
|  | MM group | 10.83 (3.97) | 0.76 | 3.62 | 2 | 145 | .03 | 0.047 |
| Mindfulness | SCM group | 13.11 (3.47) | 0.69 | 2.86 | 2 | 152 | .06 | 0.036 |
|  | MM group | 11.84 (3.53) | 0.71 | 0.17 | 2 | 144 | .84 | 0.002 |
| Over-Identification | SCM group | 12.79 (3.81) | 0.76 | 4.08 | 2 | 147 | .02 | 0.053 |
|  | MM group | 13.17 (4.14) | 0.82 | 3.96 | 2 | 144 | .02 | 0.052 |
| Perceived Stress | SCM group | 25.03 (9.88) | 0.53 | 6.48 | 2 | 145 | .002 | 0.082 |
|  | MM group | 26.98 (10.42) | 0.69 | 1.41 | 2 | 145 | .25 | 0.019 |
| Work Engagement | SCM group | 28.91 (13.99) | 0.89 | 1.06 | 2 | 145 | .35 | 0.014 |
|  | MM group | 25.58 (13.63) | 0.82 | 0.45 | 2 | 145 | .64 | 0.006 |
| Vigor | SCM group | 8.60 (4.94) | 0.81 | 0.97 | 2 | 150 | .38 | 0.013 |
|  | MM group | 7.83 (4.65) | 0.79 | 0.90 | 2 | 145 | .41 | 0.012 |
| Dedication | SCM group | 10.71 (4.63) | 0.88 | 1.53 | 2 | 149 | .22 | 0.020 |
|  | MM group | 9.73 (4.49) | 0.80 | 0.75 | 2 | 146 | .47 | 0.010 |
| Absorption | SCM group | 9.60 (5.04) | 0.86 | 1.28 | 2 | 147 | .28 | 0.017 |
|  | MM group | 8.14 (5.02) | 0.75 | 0.32 | 2 | 147 | .73 | 0.004 |
| Psychological Safety | SCM group | 35.86 (7.37) | 0.73 | 3.18 | 2 | 143 | .04 | 0.043 |
|  | MM group | 33.79 (7.91) | 0.74 | 0.45 | 2 | 147 | .64 | 0.006 |
| Creativity | SCM group | 12.19 (4.57) | 0.79 | 0.77 | 2 | 147 | .46 | 0.010 |
|  | MM group | 11.44 (4.37) | 0.75 | 1.03 | 2 | 146 | .36 | 0.014 |
