# Supplementary material for: Effects of Self-Compassion and Mindfulness Interventions on Mental Health and Work-Related Outcomes Among Japanese Workers: Randomized Controlled Trial
Source: J Med Internet Res. 2026 Mar 17;28:e79991. doi: 10.2196/79991 (PMC12994763; doi:10.2196/79991)
Supplement: Multimedia Appendix 3 [file jmir-v28-e79991-s003.docx]

Appendix Table 3. Main effects for time and interaction effects for group × time using LMM

|  | ICC | Time | | | | | Interaction effects | | | | |
| --- | --- | --- | --- | --- | --- | --- | --- | --- | --- | --- | --- |
|  |  | F value | df1 | df2 | *P*-value | Partial *η*^2^ | F value | df1 | df2 | *P*-value | Partial *η*^2^ |
| Psychological Distress | 0.64 | 1.15 | 2 | 296 | .32 | 0.008 | 0.58 | 2 | 296 | .56 | 0.004 |
| Work Performance | 0.57 | 5.52 | 2 | 292 | .00 | 0.036 | 3.23 | 2 | 293 | .04 | 0.022 |
| Work Inefficiency | 0.39 | 1.67 | 2 | 286 | .19 | 0.012 | 0.97 | 2 | 287 | .38 | 0.007 |
| Cognitive Flexibility | 0.76 | 2.49 | 2 | 280 | .09 | 0.017 | 1.81 | 2 | 280 | .17 | 0.013 |
| Self-Compassion | 0.84 | 3.93 | 2 | 267 | .02 | 0.029 | 0.96 | 2 | 267 | .39 | 0.007 |
| Self-Kindness | 0.78 | 1.35 | 2 | 290 | .26 | 0.009 | 1.25 | 2 | 290 | .29 | 0.009 |
| Self-Judgment | 0.77 | 3.84 | 2 | 293 | .02 | 0.025 | 1.34 | 2 | 293 | .26 | 0.009 |
| Common Humanity | 0.77 | 1.88 | 2 | 292 | .15 | 0.013 | 0.20 | 2 | 292 | .82 | 0.001 |
| Isolation | 0.73 | 1.04 | 2 | 294 | .35 | 0.007 | 0.18 | 2 | 294 | .84 | 0.001 |
| Mindfulness | 0.71 | 2.01 | 2 | 296 | .14 | 0.013 | 1.15 | 2 | 296 | .32 | 0.008 |
| Over-Identification | 0.79 | 2.02 | 2 | 291 | .13 | 0.014 | 1.27 | 2 | 291 | .28 | 0.009 |
| Perceived Stress | 0.62 | 3.35 | 2 | 290 | .04 | 0.023 | 1.10 | 2 | 290 | .34 | 0.008 |
| Work Engagement | 0.86 | 0.49 | 2 | 289 | .61 | 0.003 | 0.30 | 2 | 289 | .74 | 0.002 |
| Vigor | 0.80 | 0.25 | 2 | 294 | .78 | 0.002 | 0.05 | 2 | 295 | .95 | 0.000 |
| Dedication | 0.84 | 0.64 | 2 | 295 | .53 | 0.004 | 0.43 | 2 | 295 | .65 | 0.003 |
| Absorption | 0.81 | 1.25 | 2 | 293 | .29 | 0.008 | 1.15 | 2 | 293 | .32 | 0.008 |
| Psychological Safety | 0.74 | 2.04 | 2 | 291 | .13 | 0.014 | 1.26 | 2 | 290 | .29 | 0.009 |
| Creativity | 0.78 | 1.49 | 2 | 293 | .23 | 0.010 | 1.78 | 2 | 293 | .17 | 0.012 |
